# Differential Protumoral Mechanisms Induced by CAFs in Cervical Cancer Cells Occur Independently of 17β-Estradiol Stimulation

**DOI:** 10.3390/cancers18101509

**Published:** 2026-05-08

**Authors:** Jonathan René García-Bernal, Luis Javier Reséndiz-Castillo, Marcela Guadalupe Martínez-Barajas, Carlos Daniel Díaz-Palomera, Adrián Ramírez-de-Arellano, Alejandra Natali Vega-Magaña, Lesly Jazmin Bueno-Urquiza, Luis Reneé González-Lucano, Marcela Peña-Rodríguez, Julio César Villegas-Pineda, Ana Laura Pereira-Suárez

**Affiliations:** 1 Instituto de Investigación en Cáncer e Infecciones, Centro Universitario de Ciencias de la Salud, Universidad de Guadalajara, Guadalajara 44340, Jalisco, Mexico; jonathan.garcia6111@alumnos.udg.mx (J.R.G.-B.); luis.resendiz9893@alumnos.udg.mx (L.J.R.-C.); marcela.martinez@academicos.udg.mx (M.G.M.-B.); daniel.diaz@academicos.udg.mx (C.D.D.-P.); adrian.ramirez@academicos.udg.mx (A.R.-d.-A.); alejandra.vega@academicos.udg.mx (A.N.V.-M.); lesly.bueno@academicos.udg.mx (L.J.B.-U.); marcela.pena@academicos.udg.mx (M.P.-R.); julio.villegas@academicos.udg.mx (J.C.V.-P.); 2 Escuela de Ciencias de la Salud y Medicina, Instituto Tecnológico y de Estudios Superiores de Monterrey, Campus Guadalajara, Zapopan 45138, Jalisco, Mexico; rene.lucano@tec.mx; 3 Departamento de Microbiología y Patología, Centro Universitario de Ciencias de la Salud, Universidad de Guadalajara, Guadalajara 44340, Jalisco, Mexico

**Keywords:** cancer-associated fibroblasts, cervical cancer, estradiol, metabolism, migration, pro-tumoral effect

## Abstract

Background: E2 is fundamental to the onset and progression of CC. CAFs are the most abundant stromal population in the tumor microenvironment. CAFs have been shown to promote protumoral effects in CC cell lines; however, the effects of E2-stimulated CAFs remain to be explored. Objectives: This study aimed to evaluate whether E2-stimulated CAFs modify the behavior of SiHa and HeLa tumor cells, including their metabolism, ROS production, migration, apoptosis, and gene expression. Methods: Characterization and estrogen receptor expression in primary CAF cultures were evaluated by immunofluorescence. Supernatants from non-stimulated and E2-stimulated CAFs were used to culture CC cells, and mitochondrial metabolism was measured by MTT, ROS production by H2DCFDA, migration using the wound-healing assay, apoptosis by Annexin V assay, and gene expression by next-generation RNA-seq. Results: Stimulation of CC cells with CAFs’ supernatants demonstrated that it influences tumor cells in different ways. In SiHa cells, the metabolic shift was supported by decreased mitochondrial metabolism, increased ROS production, and enhanced gene enrichment for glycolysis and hypoxia responses. On the other hand, in HeLa cells, CAFs enhanced migration and gene enrichment in KRAS signaling, epithelial-mesenchymal transition, and the proinflammatory response, via enrichment of the IL6/JAK/STAT3 signaling pathway, along with the overexpression of cytokines such as IFN-γ, IL-6, and IL-8. Conclusions: CAFs exert protumoral effects by promoting a metabolic shift in SiHa cells or enhancing migration and cytokine secretion in HeLa cells; these effects are independent of E2 stimulation in CAFs.

## Simple Summary

Chronic exposure to estrogens, particularly 17β-estradiol (E2), is a significant risk factor in cervical cancer (CC) pathogenesis, partly by promoting metabolic reprogramming and enhancing tumor cell survival. Within the tumor microenvironment, cancer-associated fibroblasts (CAFs) represent the predominant stromal component and interact dynamically with immune and neoplastic cells, as well as cytokines, growth factors, and hormones that collectively facilitate tumor progression. Although the protumorigenic influence of CAFs on CC cell lines is established, the specific impact of E2-stimulated CAFs on tumor cell behavior remains unclear. In this study, we evaluated the effects of E2-stimulated CAFs on metabolic activity, reactive oxygen species (ROS) generation, migratory capacity, apoptosis, and gene expression profiles. Notably, E2-stimulated CAFs produced effects comparable to non-stimulated CAFs, with similar responses in tumor cells. These findings indicate that CAF-mediated tumor-promoting functions in CC are maintained independently of E2 stimulation, underscoring their intrinsic capacity to drive tumor progression.

## 1. Introduction

Cervical Cancer (CC) is recognized as the fourth most prevalent neoplasm among women, with over 660,000 new cases and approximately 350,000 fatalities reported by the year 2022 [1]. This carcinoma arises at the junction between stratified squamous and columnar epithelium, known as the squamocolumnar junction or transformation zone. It is here that infection by high-risk oncogenic viruses, such as Human Papillomavirus (HPV)-16 and HPV-18, via the E6 and E7 oncoproteins leads to neoplastic transformation [2].

17β-estradiol (E2) serves as a pivotal factor in the pathogenesis of CC, as prolonged exposure to this hormone facilitates not only its initiation but also its maintenance and progression [3]. This hormone exerts its effects through the nuclear Estrogen Receptors (ERs), ERα and ERβ, or the G protein-coupled estrogen receptor (GPER), eliciting distinct effects, such as metabolic activation [4] or cell death in target cells [5]. These ERs are expressed in healthy cervical tissue; however, their expression levels increase in malignant lesions, mediating distinct effects depending on which ER is activated in tumor cells, promoting the Warburg effect [6], senescence, and apoptosis [5].

CC cells coexist with various cellular and molecular components within the Tumor Microenvironment (TME). The TME includes not only tumor and immune cells but also stromal cells, such as cancer-associated fibroblasts (CAFs), extracellular matrix (ECM) proteins, cytokines, and hormones such as E2 [7,8]. CAFs constitute the most abundant stromal population within the TME, contributing to ECM remodeling and facilitating tumor progression [9]. These cells express a variety of markers, specifically vimentin, alpha-Smooth Muscle Actin (α-SMA), and Fibroblast Activation Protein (FAP), which have been associated with pro-tumoral effects such as migration, proliferation, and metabolic alterations in tumor cells [10,11].

CAFs exert a variety of functions in the TME; particularly in CC, they facilitate tumor progression in HeLa cells via Transforming Growth Factor-β1 (TGF-β1) and Stromal Cell-Derived Factor 1 (SDF-1) [12]. Notwithstanding these findings, the impact of E2 mediated by CAFs remains unexamined. Accordingly, the present study investigates the influence of E2-stimulated CAF supernatants on essential cellular processes related to tumor progression, including metabolism, migration, apoptosis, and gene expression, within cervical cancer cell models.

## 2. Materials and Methods

### 2.1. Cell Culture

CC cell lines SiHa and HeLa were obtained from the American Type Culture Collection (ATCC, Manassas, VA, USA). Both cell lines were cultured in DMEM Glutamax (Cat. 10566-016, Gibco, Waltham, MA, USA) supplemented with 10% heat-inactivated fetal bovine serum (FBS, Cat. 26140079, Gibco, Waltham, MA, USA) and 1% antibiotic-antimycotic solution (Cat. 15240062, Gibco, Waltham, MA, USA) under standard conditions (humidified atmosphere with 5% CO_2_).

### 2.2. CAFs Isolation and Culture

Primary cultures of CAFs were derived from two different lesions of cervical cancer, one from low-grade (IB2, identified as LG-CAF) and another from high-grade (IIIC2, identified as HG-CAF). Briefly, biopsies were minced into ~1 mm^3^ pieces and disaggregated using Dispase (Cat. 17105-041, Gibco, Tokyo, Japan), Collagenase (Cat. 17018-029, Gibco, Waltham, MA, USA), and Trypsin/EDTA (Cat. 25200072, Gibco, Waltham, MA, USA). After enzymatic digestion, the tissue was cultured for 2 h in a culture flask containing DMEM/F12 Glutamax (Cat. 10565-018, Gibco, Waltham, MA, USA) supplemented with 10% FBS and 1% antibiotic-antimycotic. Subsequently, multiple washes with PBS were conducted to remove non-adherent cells or tissue remnants, following which enrichment medium was added to the flask. The attached cells were then cultured in DMEM/F12 Glutamax supplemented with 10% FBS and 1% antibiotic-antimycotic until reaching a high confluence, in preparation for further characterization to confirm the isolation of CAFs. The morphology and growth behavior of the CAFs were assessed at high confluence, and the presence of CAFs in CC tissue was confirmed through hematoxylin and eosin staining of tissue sections.

### 2.3. CAFs’ Markers and ERs Characterization

Once the CAFs culture reached 85% of confluence, 7.5 × 10^3^ cells/well were seeded into immunofluorescence slide chambers. First, cells were seeded and allowed to adhere for 24 h. Cells were then fixed with paraformaldehyde, permeabilized with Triton-X100 (Cat. 161-0407, BioRad, Hercules, CA, USA), and blocked with horse serum (Cat. 16050-114, Gibco, Waltham, MA, USA). Overnight incubation with primary antibodies was performed at 4 °C for markers: vimentin (1:200, Cat. AB8978, Abcam, Cambridge, MA, USA), α-SMA (1:500, Cat. AB7817, Abcam), and FAP (1:200, Cat. AB28244, Abcam), or for estrogen receptors: ERα (1:100, Cat. SC-8002, Santa Cruz Biotechnology, Dallas, TX, USA), ERβ (1:50, Cat. SC-373853, Santa Cruz), and GPER (1:200, Cat. AB39742, Abcam). Incubation with secondary antibodies at room temperature was performed using goat anti-mouse-AF488 (1:1000, Cat. AB150113, Abcam, Cambridge, MA, USA) and goat anti-rabbit-AF647 (1:1000, Cat. AB150083, Abcam, Cambridge, MA, USA). Nuclei were counterstained with DAPI (1:10,000, Cat. D1306, Invitrogen, Waltham, MA, USA). Slides were mounted and visualized on a confocal microscope (Leica Microsystems, Wetzlar, Germany). ER expression was evaluated using FIJI (NIH v1.54p).

### 2.4. CAFs’ Stimulation with E2 and Supernatant Collection

Once characterized, CAFs were cultured in DMEM Glutamax supplemented with 10% FBS and 1% antibiotic-antimycotic. Supernatants were collected from CAFs between passages 2 and 7. Upon reaching 85% confluence, the culture medium was replaced and supplemented with 17-β estradiol (E2) at 10 nM (Sigma-Aldrich, St. Louis, MO, USA) for 48 h, with an intermediate replenishment at 24 h. After 48 h, cells were washed multiple times with PBS, and the culture medium was replaced with DMEM Glutamax containing only 1% antibiotic-antimycotic for an additional 48 h. Finally, the supernatant was collected, filtered, and stored at −80 °C until needed, with use within 4 weeks. The group without E2 stimulation followed the same methodology but intentionally omitted E2.

### 2.5. Mitochondrial Activity Assay

The MTT assay was used to evaluate the effect of CAFs’ supernatant on mitochondrial activity in cervical cancer cell lines. Concisely, 5 × 10^3^ cells/well were cultured in 96-well plates and deprived of FBS for 24 h. After this, cancer cells were cultured with CAFs’ supernatant for 24 h. The MTT reagent (5 mg/mL, Cat. M5655-500MG, Sigma-Aldrich, St. Louis, MO, USA) was then added, and the cells were incubated for 4 h in the dark under standard culture conditions. Finally, the supernatant was discarded, and formazan crystals were dissolved in DMSO (Cat. 472301-100ML, Sigma-Aldrich, St. Louis, MO, USA). Absorbance was measured at 570 nm using the Multiskan GO plate reader (Thermo Scientific, Waltham, MA, USA). Three independent experiments were conducted in triplicate.

### 2.6. ROS Production Assay

The fluorometric DCFDA/H2DCFDA assay was used to evaluate ROS production induced by stimulation of CAF supernatant. Shortly, 5 × 10^4^ cells/well were cultured in 96-well plates and deprived of FBS for 24 h. Later, cervical cancer cells were stimulated with CAF supernatants for 24 h to assess ROS production using the DCFDA/H2DCFDA assay (Cat. AB113851, Abcam, Cambridge, MA, USA) per the manufacturer’s instructions. Three independent experiments were performed in triplicate.

### 2.7. Migration Assay

To determine whether CAF supernatant induces tumor cell migration, a wound-healing assay was conducted. First, cells were cultured in a 6-well plate until reaching 85% confluence. Cells were treated with Mitomycin-C (0.005 mg/mL; Cat. Y0000378, Sigma-Aldrich, St. Louis, MO, USA) for 2 h, then discarded. Subsequently, a scratch was made across the middle of each well using a 200 μL tip. Cell debris was removed by washing with PBS, and cells were cultured in CAFs’ supernatant for 24 h. Photographs were taken at time 0 and after 24 h, at the same point in each well, to document tumor cell migration. The percentage of covered area was calculated using the following equation: % of covered area = [(Initial % − final %)/Initial %] × 100%, with data obtained using the wound-healing plugin published by Suarez-Arnedo et al. [13]. Three independent experiments were conducted in duplicate.

### 2.8. Apoptosis Assay

To determine whether CAFs can affect tumor cell apoptosis, Annexin V/PI staining was performed. Briefly, cells were cultured in 24-well plates with CAFs’ supernatant, with or without cisplatin (80 μg/mL), for 24 h. Subsequently, cells were harvested and stained with the Annexin V/PI kit (Cat. V13245, Invitrogen, Waltham, MA, USA) according to the manufacturer’s instructions. Three independent experiments were conducted.

### 2.9. Cytokine Production Assay

To determine the basal production of cytokines by CAFs and tumor cells stimulated with CAFs’ supernatants, a multiplex immunoassay experiment was performed. The CAFs’ supernatant was obtained as previously described in Section 2.4. For SiHa and HeLa, the cells were stimulated in a 6-well plate with CAFs’ supernatant for 24 h, washed multiple times with PBS to avoid cross-contamination from CAFs’ supernatant, and then cultured in DMEM without FBS for an additional 24 h to collect cytokines produced by tumor cells. The tumor cells’ supernatant was immediately stored at −80 °C until use. Cytokine concentrations were measured using a Bio-Plex Pro Human Cytokine 8-Plex Panel (TNF-α, IFN-γ, GM-CSF, IL-2, IL-4, IL-6, IL-8, and IL-10, Cat. M50000007A, Bio-Rad, Hercules, CA, USA) according to the manufacturer’s instructions. Data was obtained as pg/mL. Determination was conducted in three independent experiments.

### 2.10. RNA Extraction and RNA-Seq Analysis

To identify the genomic effects of CAFs’ supernatant on tumor cell lines, RNA-seq analysis was performed. Briefly, 4 × 10^6^ cells of each cervical cancer cell line were stimulated with HG CAF’s supernatants for 24 h, and RNA was extracted using the RNeasy Plus Mini Kit (Cat. 74634, Qiagen, Venlo, The Netherlands) according to the manufacturer’s instructions. RNA concentration was determined by measuring absorbance at 260/280 nm and was preserved at −80 °C in GenTegra RNAssure elution tubes (Cat. GTR50-LQ, GenTegra, Pleasanton, CA, USA) until further processing. RNA next-generation sequencing was performed on the NovaSeq 6000 platform managed by Novogene Bioinformatics Technology Co., Ltd. (Sacramento, CA, USA). Raw data were analyzed on the open-source Galaxy platform (v0.12.1) using FastQC (v0.12.1) for quality control. Differentially expressed genes (DEGs) were analyzed in RStudio (R v4.5.2) using the DESeq2 tool (v1.50.2).

### 2.11. Gene Set Enrichment Analysis

Gene Set Enrichment Analysis (GSEA) was performed using the preranked algorithm. A preranked file based on Wald’s statistics was generated from DESeq2 results and loaded into the Broad Institute’s software. The analysis used the Hallmark collection gene set from the Molecular Signatures Database (h.all.v2026.1.Hs.symbols.gmt). Enriched pathways were considered significant if NES > 1.5 and FDR < 0.25. Bubble plots were generated for the top 10 enriched pathways. Heatmaps were generated by selecting leading-edge genes contributing to hallmark enrichment, filtering for *p*-adjusted values < 0.05 and log2FoldChange > 1.5, and categorizing them into pathways based on hallmark similarity.

### 2.12. Statistics

Statistical analyses were performed using GraphPad 9.0.0. All experiments were conducted with at least three independent replicates. Data normality was assessed. Results were analyzed using the appropriate test: Student’s *t*-test, Kruskal–Wallis, or one-way ANOVA. A *p*-value < 0.05 was considered statistically significant.

## 3. Results

Given that CAFs are the second most abundant cell population in the TME and that prolonged exposure to hormones, such as E2, is a crucial factor in the development of CC, we investigated the effect of E2-stimulated CAFs on the behavior of CC tumor cells. To conduct this study, primary CAFs from low- and high-grade lesions were stimulated with E2, and the resulting supernatant was used to culture SiHa and HeLa CC cell lines for functional analyses. Subsequently, metabolism, reactive oxygen species (ROS), migration, apoptosis, cytokine production, and gene expression were evaluated in the cell lines.

### 3.1. Characterization of the Primary Culture of CAFs

**Figure 1:**
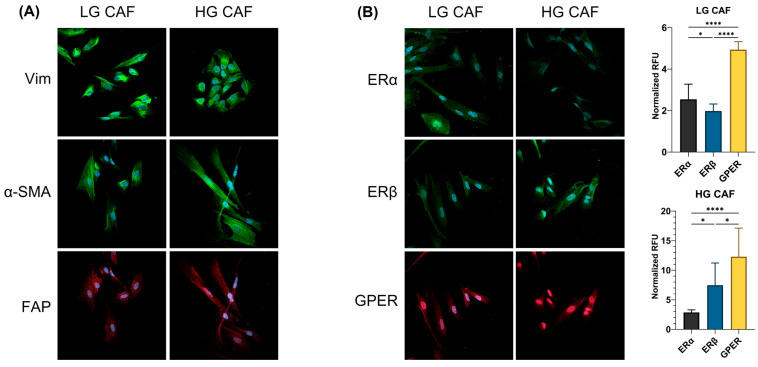
Characterization of CAF markers and expression of ERs in primary culture of fibroblasts from low- and high-grade cancer lesions. Expression of CAF markers and ERs was evaluated by immunofluorescence using an Alexa Fluor 488 (green) or Alexa Fluor 647 (red) conjugated secondary antibody and nuclear staining with DAPI (blue). Merged images are shown at 40× magnification. (**A**) Expression of vimentin, α-SMA, and FAP in primary culture of LG and HG CAFs. (**B**) Expression of ERα, ERβ, and GPER in primary culture of LG and HG CAFs. Quantification of relative fluorescence units (RFU) was performed with FIJI and normalized with DAPI. RFU was calculated from at least 10 different images for each ER. Statistical analysis was performed using one-way ANOVA. Data is presented as mean ± SD. * *p* < 0.05, **** *p* < 0.0001. LG CAF: Cancer-associated fibroblasts from low-grade lesion, HG CAF: Cancer-associated fibroblasts from high-grade lesion, Vim: Vimentin, α-SMA: Alpha smooth muscle actin, FAP: Fibroblast activation protein, ERα: Estrogen receptor alpha, ERβ: Estrogen receptor beta, GPER: G protein-coupled estrogen receptor.

CAFs were isolated from CC biopsies, one from a low-grade and another from a high-grade lesion, according to the International Federation of Gynecology and Obstetrics (FIGO) classification. Tissue sections were assessed to confirm the presence of CAFs within tumor biopsies, as indicated by eosinophilic staining in the stroma (Supplementary Figure S1). Furthermore, macroscopical evaluation revealed that CAFs displayed non-uniform directionality, stellate morphology, and overlapping growth (Supplementary Figure S2) as previously reported by Xiao and colleagues [12]. To conclusively establish appropriate CAF isolation, immunofluorescence was used to evaluate the expression of vimentin, α-SMA, and FAP, which are the main markers of the CAF lineage and activation (Figure 1A). All CAFs from both biopsies expressed these proteins, indicating the presence of activated CAFs. As expected, vimentin and α-SMA were located in the cytoplasm, and FAP in the cell membrane.

### 3.2. Primary Culture of CAFs from CC Biopsies Expresses Strong Amounts of GPER

After characterization, expression of ERs was assessed in primary culture of CAFs obtained from CC biopsies by immunofluorescence. Interestingly, CAFs expressed the ERs at different levels. GPER expression was significantly higher (*p* < 0.0001) than the expression of ERα and ERβ. In low-grade CAFs (LG CAF), ERα is present in a greater proportion than ERβ (*p* < 0.05), in contrast to what is observed in high-grade CAFs (HG CAF), where the expression of ERβ is approximately twofold that of ERα (*p* < 0.05) (Figure 1B).

### 3.3. CAFs Decrease the Mitochondrial Metabolism in SiHa but Not in HeLa Cells

**Figure 2:**
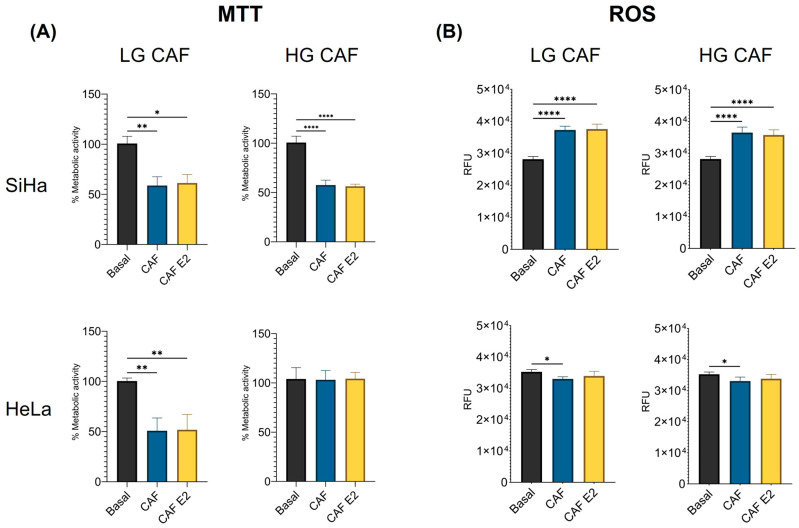
CAFs decrease the mitochondrial metabolism and enhance the ROS production in SiHa cells. Mitochondrial metabolism was assessed by MTT assay, and ROS by H2DCFDA. SiHa and HeLa cells were cultured with LG or HG CAFs’ supernatant prior to metabolism or ROS measurement. (**A**) Mitochondrial metabolism of SiHa and HeLa cells stimulated with LG and HG CAFs, and metabolic activity was normalized against the control. (**B**) Production of ROS by SiHa and HeLa cells stimulated with LG and HG CAFs. Statistical analysis was first performed with a normality test using the Shapiro–Wilk test and later with Kruskal–Wallis or one-way ANOVA. Data is presented as mean ± SD. * *p* < 0.05, ** *p* < 0.01, **** *p* < 0.0001. LG CAF: Cancer-associated fibroblasts from low-grade lesion, HG CAF: Cancer-associated fibroblasts from high-grade lesion, Basal: Cells cultured with DMEM, CAF basal: Cells cultured with CAF’s supernatant without E2 stimulation, CAF E2: Cells cultured with CAF’s supernatant with previous stimulation with E2.

One of the main characteristics of cancer is the dysregulation of cellular metabolism. Within our research group, it was demonstrated that E2 increases the metabolism of HeLa and SiHa cells. To evaluate the effect of CAFs obtained from CC biopsies with and without E2 stimulation on the mitochondrial metabolism of these cell lines, we performed MTT and ROS assays. Both LG CAF and HG CAF, with and without E2, significantly decreased the metabolism of SiHa cells (LG: CAF: *p* < 0.01, CAF E2 *p* < 0.05; HG: CAF *p* < 0.0001, CAF E2 *p* < 0.0001). A similar result was obtained only in HeLa cells treated with LG CAFs’ supernatant (CAF *p <* 0.01, CAF E2 *p* < 0.01); HG CAFs did not alter metabolism in these cell lines, which remained similar to basal levels. The effect of CAFs on cancer cells was not related to the biopsy stage from which they were obtained. It is important to note that, in all cases, E2 stimulation of CAFs did not influence their impact on tumor cell metabolism. (Figure 2A). When evaluating ROS, we observed that supernatants from LG CAFs and HG CAFs, with or without E2 stimulation, significantly increased ROS production in SiHa cells. (*p* < 0.0001), whereas in HeLa cells, the supernatant from non-E2-stimulated CAFs decreased it (*p* < 0.05) (Figure 2B). The impact of these CAFs on ROS production in tumor cells was unaffected by the presence of E2.

### 3.4. CAFs Promote Cell Migration in HeLa but Not in SiHa Cells

**Figure 3:**
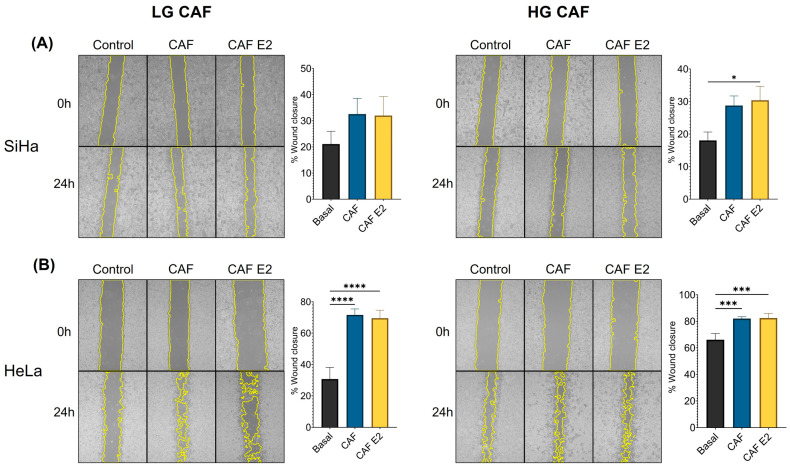
CAFs promote cell migration in HeLa cells. Migration was assessed using a wound-healing assay and quantified as the percentage of wound closure. (**A**) Migration of SiHa cultured with LG and HG cervical cancer lesion CAFs’ supernatant. (**B**) Migration of HeLa cultured with LG and HG cervical cancer lesion CAFs’ supernatant. In photographs, the yellow color is used to highlight the measured % of area in the assay. Statistical analysis was performed first with a normality test using the Shapiro–Wilk test and later using Kruskal–Wallis for panel A and one-way ANOVA for panel B. Data are presented as mean ± SD. * *p* < 0.05, *** *p* < 0.001, **** *p* < 0.0001. LG CAF: Cancer-associated fibroblasts from low-grade lesion, HG CAF: Cancer-associated fibroblasts from high-grade lesion, Basal: Cells cultured with DMEM, CAF: Cells cultured with CAF’s supernatant without E2 stimulation, CAF E2: Cells cultured with CAF’s supernatant with previous stimulation with E2.

Another characteristic of tumor cells is their capacity to migrate and invade other tissues. To ascertain whether CAFs derived from CC, with or without E2 stimulation, influence tumor cell migration, wound closure assays were conducted. LG and HG CAFs generally tended to enhance SiHa cell migration, with only the E2-stimulated HG CAFs demonstrating a statistically significant increase (*p* < 0.05) (Figure 3A). Conversely, LG and HG CAFs considerably promoted the migration of HeLa cells. (*p* < 0.0001 and *p* < 0.001, respectively) (Figure 3B). This increase is not associated with the extent of injury in the biopsies from which CAFs were derived, nor with the presence of E2.

### 3.5. CAFs Do Not Affect the Apoptosis in CC Cell Lines

**Figure 4:**
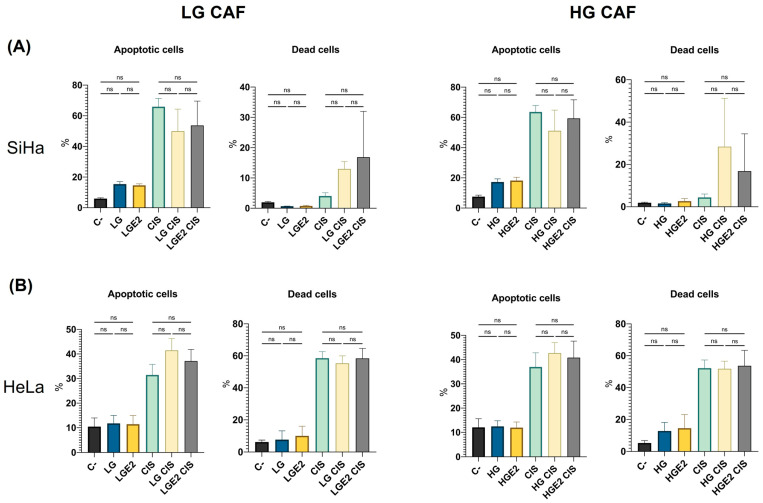
CAFs do not affect apoptosis in CC cell lines. Apoptosis was assessed by Annexin V staining for phosphatidylserine exposure, and cell death by PI staining, with results expressed as percentages. (**A**) Apoptosis and death of SiHa cultured with low- and high-grade cervical cancer lesion CAFs’ supernatant. (**B**) apoptosis and death of HeLa cultured with low- and high-grade cervical cancer lesion CAFs’ supernatant. Statistical analysis was first performed with a normality test using the Shapiro–Wilk test, followed by Kruskal–Wallis or one-way ANOVA. Data is presented as mean ± SD. ns: Not significant. C-: Negative control, only cultured with DMEM, LG: Cancer-associated fibroblasts from low-grade lesion, LGE2: Cancer-associated fibroblasts from low-grade lesion with previous E2 stimulation, CIS: Cisplatin 80 μg/mL, LG CIS: Cancer-associated fibroblasts from low-grade lesion + cisplatin 80 μg/mL, LGE2 CIS: Cancer-associated fibroblasts from low-grade lesion with previous E2 stimulation + cisplatin 80 μg/mL, HG: Cancer-associated fibroblasts from high-grade lesion, HGE2: Cancer-associated fibroblasts from high-grade lesion with previous E2 stimulation, HG CIS: Cancer-associated fibroblasts from high-grade lesion + cisplatin 80 μg/mL, HGE2 CIS: Cancer-associated fibroblasts from high-grade lesion with previous E2 stimulation + cisplatin 80 μg/mL.

Tumor cells have been demonstrated to resist apoptosis, a hallmark that is very well defined. This characteristic is regulated by CAFs, which help protect cells from chemotherapy-induced cell death. To determine whether CAFs, with or without E2 stimulation, induce tumor cell apoptosis and death, the proportions of cells undergoing these processes were assessed. The supernatant derived from CAFs did not influence apoptosis or cell death in SiHa (Figure 4A) and HeLa cells (Figure 4B), as the findings were not statistically significant. This outcome is comparable to that observed following cisplatin treatment, given that the percentages of SiHa and HeLa cells undergoing apoptosis and cell death do not vary significantly.

### 3.6. CAFs Induce the Expression of Proinflammatory Cytokines in HeLa Cells

**Figure 5:**
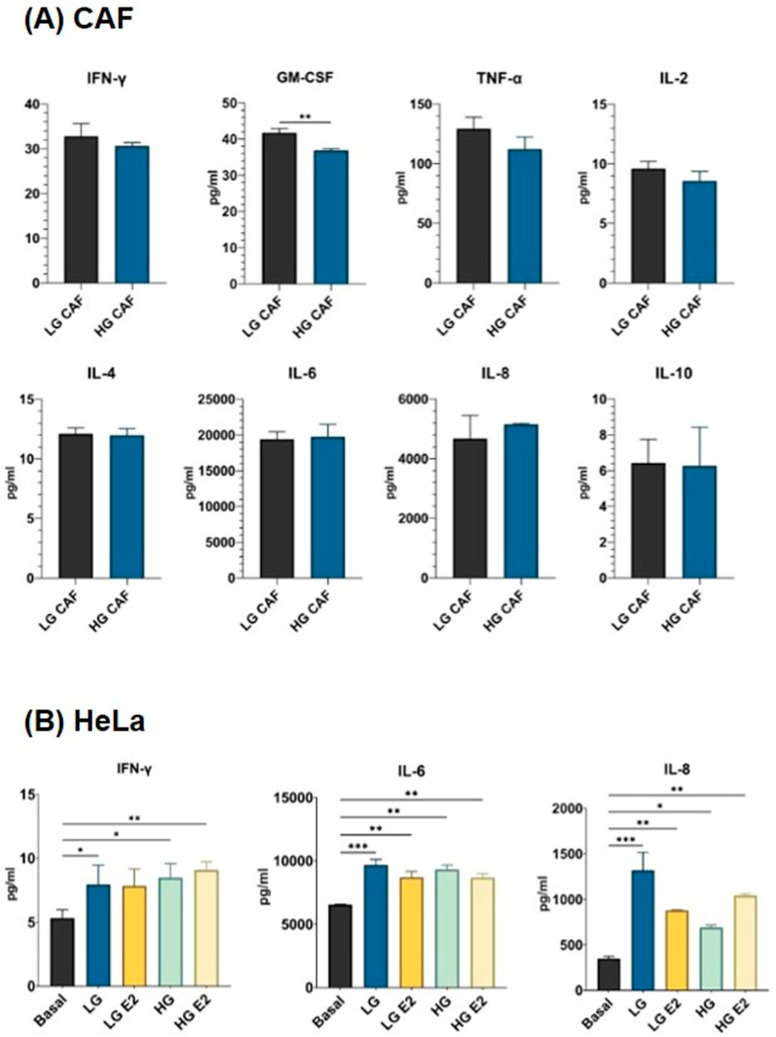
Cytokines produced by CAFs and the HeLa cell line stimulated with CAFs’ supernatant. Cytokine production in the supernatant of CAFs or the HeLa cervical cancer cell line was evaluated using a multiplex assay. (**A**) Cytokine production of CAFs from low- and high-grade cervical cancer lesions. (**B**) Production of cytokines by the HeLa cell line stimulated with CAFs supernatant. Statistical analysis was performed with Student’s T test for CAFs, for cell lines first with a normality test using the Shapiro–Wilk test, and later using Kruskal–Wallis or one-way ANOVA. Data is presented as mean ± SD. * *p* < 0.05, ** *p* < 0.01, *** *p* < 0.001. LG CAF: Cancer-associated fibroblasts from low-grade lesion, HG CAF: Cancer-associated fibroblasts from high-grade lesion, Basal: Cells cultured with DMEM, LG: Cells cultured with low-grade CAFs supernatant, LG E2: Cells cultured with E2-stimulated CAFs supernatant, HG: Cells cultured with high-grade CAFs supernatant, HG E2: Cells cultured with E2-stimulated CAFs supernatant.

Within the context of the TME, CAFs are active cellular components that engage in diverse functions, including the modification of the ECM and the secretion of cytokines that influence immune responses and tumor cell dynamics. Consequently, we have elected to assess which cytokines are produced basally by low- and high-grade CAFs, as well as by SiHa and HeLa cells stimulated with the supernatants from these CAFs. Under basal conditions, CAFs produce cytokines in varying quantities (Figure 5A). Of the evaluated molecules, Granulocyte-Macrophage Colony-Stimulating Factor (GM-CSF, *p* < 0.01) was found to be produced in lower concentrations by HG CAFs compared to LG CAFs. No significant difference was found in the other cytokines evaluated. Although no significant variation in IL-6 and IL-8 production was observed concerning the degree of lesion origin of the CAFs, elevated concentrations of these cytokines were detected (IL-6 > 19,000 pg/mL and IL-8 > 4000 pg/mL).

Conversely, the production of cytokines by SiHa and HeLa tumor cells stimulated with the supernatant of cancer-associated fibroblasts (CAFs) was also assessed. In HeLa cells (Figure 5B), a notable increase in IFN-γ secretion was observed upon exposure to both HG CAF stimuli (*p* < 0.05). Furthermore, the production levels of IL-6 (>7500 pg/mL, *p* < 0.01) and IL-8 (>500 pg/mL, *p* < 0.05) were elevated across all conditions. Nevertheless, no statistically significant differences were detected in SiHa cells.

### 3.7. HG CAFs Promote the Enrichment of DEGs and Hallmarks in SiHa, Distinct from Those in HeLa Cells

**Figure 6:**
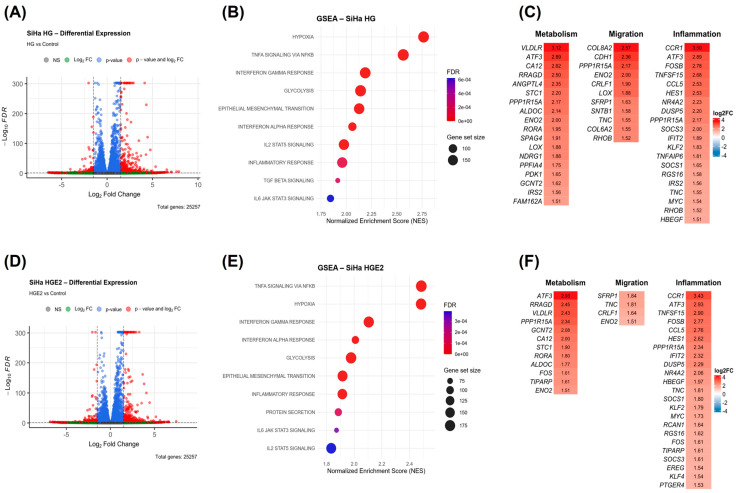
CAFs promote the enrichment of hallmarks and DEGs related to metabolic shifts in SiHa cells. Analysis was conducted on SiHa cells stimulated with either CAFs or E2-stimulated CAFs supernatant. (**A**,**D**) Volcano plot illustrating DEGs with FC ≤ −1.5 and ≥1.5, with a *p*-adjusted value of <0.05. (**B**,**E**) Bubble plot of Hallmark enrichment analysis by GSEA from Broad Institute’s gene collection with NES > 1.5 and FDR < 0.25. (**C**,**F**) Heatmaps of selected DEGs from enriched hallmarks with log2 fold change > 1.5 and *p*-adjusted value < 0.05. SiHa HG: SiHa cells with stimulation of CAFs supernatant, SiHa HGE2: SiHa cells stimulated with E2-stimulated CAFs supernatant. In volcano plots, red dots show DEGs. Bubble plot of the top 10 up-enriched pathways. Heatmaps show hallmark genes categorized by contribution to enrichment of pathway.

To evaluate the impact of CAFs on the gene expression of SiHa and HeLa cell lines, an exploratory analysis was conducted utilizing next-generation RNA sequencing on these tumor cells, stimulated solely with HG CAFs, as no functional differences were observed between LG and HG CAFs. In SiHa cells stimulated with CAF supernatants, volcano plots demonstrate a substantial number of differentially expressed genes (DEGs), primarily overexpressed (Log2FoldChange > 1.5) in both conditions (Figure 6A,D). GSEA enrichment analysis revealed the presence of enriched pathways in response to both stimuli (NES > 1.5 and *p*-adj < 0.05), including hypoxia, glycolysis, epithelial-mesenchymal transition, and inflammatory pathways associated with TNF-α signaling via NF-κB, the inflammatory response, and IL-6 signaling via JAK/STAT3. Notably, TGF-β signaling was exclusively enriched in the condition involving HG CAF (Figure 6B,E). Finally, the genes contributing to the core enrichment of these pathways are illustrated in the heatmaps in Figure 6C,F, organized by their functions in metabolism, migration, and inflammation.

**Figure 7:**
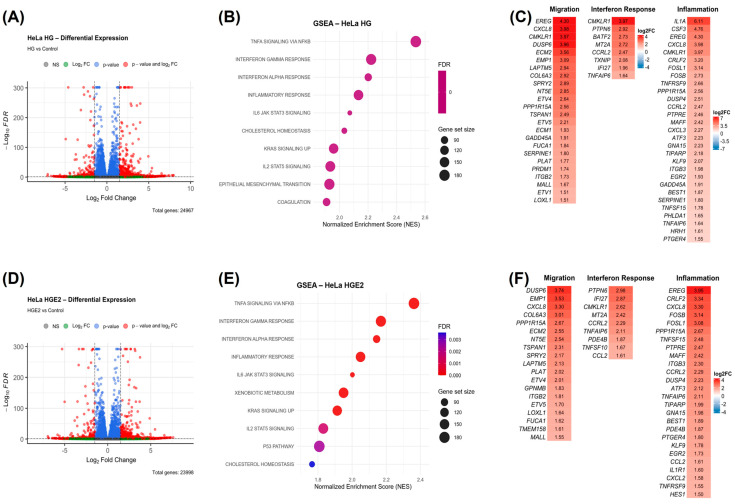
CAFs promote the enrichment of hallmarks and DEGs associated with migration and proinflammatory responses in HeLa cells. An analysis was conducted on HeLa cells stimulated with CAFs or with supernatant from E2-stimulated CAFs. (**A**,**D**) Volcano plot illustrating DEGs with FC ≤ −1.5 and ≥1.5, with a *p*-adjusted value of <0.05. (**B**,**E**) Bubble plot of Hallmark enrichment analysis by GSEA from Broad Institute’s gene collection with NES > 1.5 and FDR < 0.25. (**C**,**F**) Heatmaps of selected DEGs from enriched hallmarks with log2 fold change > 1.5 and *p*-adjusted value < 0.05. HeLa HG: HeLa cells with stimulation of CAFs supernatant, HeLa HGE2: HeLa cells stimulated with E2-stimulated CAFs supernatant. In volcano plots, red dots show DEGs. Bubble plot of the top 10 up-enriched pathways. Heatmaps show hallmark genes, categorized by their contribution to pathway enrichment.

Furthermore, in HeLa cells stimulated with HG CAFs supernatants, we also observed that volcano plots predominantly indicated a substantial number of overexpressed DEGs (Figure 7A,D). GSEA analysis revealed enriched pathways shared by both stimuli (Figure 7B,E; NES > 1.5 and *p*-adj < 0.05), including TNF-α signaling via NF-κB, the inflammatory response, IL-6 signaling via JAK/STAT3, and the interferon response (IFN-γ and IFN-α). Additionally, KRAS signaling, related to cell migration, was identified. Notably, the enrichment of the epithelial-mesenchymal transition was exclusively observed in the group stimulated with HG CAFs. The genes contributing to the core enrichment of these pathways are categorized based on their association with migration, interferon response, and inflammation (Figure 7C,F).

## 4. Discussion

Cancer is a disease characterized by the uncontrolled proliferation of cells with abnormal features that extend beyond their typical boundaries and may invade neighboring tissues or other organs. This latter phenomenon, known as metastasis, is the primary cause of cancer-related death worldwide [14]. In women, CC is the fourth most prevalent malignancy. Despite the implementation of various strategies, including vaccination against HPV, the incidence of this form of cancer continues to rank among the top four cancers affecting women [15]. The CC results from the interplay of multiple factors, including HPV infection and the activity of E2, which establish a conducive environment for tumor development, as E2 has been demonstrated to be essential for the development of cervical tumor lesions [3].

The development of this cancer is driven by a complex network of interactions within the TME, which includes molecules such as growth factors, cytokines, ECM components, and hormones, including E2. The TME also encompasses diverse cell populations, notably tumor cells, immune cells, and CAFs, which are recognized as the primary regulators of the TME [10,16,17], playing a significant role in tumor progression and adverse responses to various therapeutic strategies, thereby establishing CAFs as a critical therapeutic target [18].

Previous studies have demonstrated the presence of ERα, ERβ, and GPER in CC tumor tissue and the effects of E2 on the metabolism of SiHa and HeLa cell lines [4]. Conversely, activation of GPER with its specific agonist G1 has been shown to decrease proliferation and mitochondrial permeability, which was associated with increased apoptosis in SiHa and HeLa cells [5]. Furthermore, it has been demonstrated that in SiHa, it increases the expression of genes related to apoptosis [19]. This supports the idea that ERα and ERβ may promote protumor effects, whereas GPER may be associated with antitumor effects. However, further studies are needed to clarify the role of each ER in the context of CC.

Within the TME, CAFs, which are highly important in regulating tumor progression, coexist with tumor cells; they promote ECM remodeling, migration, invasion, and chemoresistance, among other effects, through cross-communication between CAFs and tumor cells [20,21]. Prior studies have shown that cervical tumor cells induce a CAF-like phenotype in Mesenchymal Stem Cells (MSCs) by upregulating the expression of FAP, a characteristic marker of CAF activation. FAP has also been correlated with proliferation markers in CC tissue [10], reinforcing the role of CAFs in promoting tumor aggressiveness. Moreover, CAFs from CC have been shown to promote the proliferation, migration, and invasion of HeLa cells by overproducing Transforming Growth Factor β-1 (TGF-β-1) and Stromal cell-derived Factor-1 (SDF-1), an effect that can be reversed by adding antibodies directed against these molecules [12].

Although studies directly link the protumoral activity of CAFs in CC, the potential effect of E2 on the CAF-tumor cell communication axis has not been considered. Therefore, the main objective of this study was to determine whether E2-stimulated CAFs modify their protumoral effect in CC.

In this study, all three ERs were detected in CAFs from low- and high-grade tumors, with higher GPER expression in both. Evidence indicates that CAFs express this receptor; Luo et al. demonstrated that GPER is expressed in breast cancer CAFs and that its stimulation with the selective agonist G-1 enhances their proliferation, an effect mediated by the EGFR/ERK axis, since it is known that GPER transactivates and signals through this receptor [22]; however, the effect of E2 through CAFs remains unclear.

Communication between CAFs and tumor cells strengthens the network of interactions within the TME. Data indicate that stromal cells participate in breast cancer progression, and CAFs undergo metabolic reprogramming characterized by increased expression of glycolysis genes such as *PKM*, *HK2*, and *SLC2A1*, which is reflected in increased activity of this metabolic pathway. Additionally, it was shown that the resulting lactate was used by breast tumor cells as a fuel to activate their own metabolism [23]. Our findings reveal that the CC SiHa cell line exhibits reduced mitochondrial metabolic activity after stimulation by CAFs, which aligns with findings reported by Riera-Leal et al., who demonstrated that decreased mitochondrial permeability is associated with dysfunction and promotes the activation of glycolysis, as evidenced by increased glucose consumption and lactate production [6].

Mitochondria are a major source of ROS, which are produced in the Electron Transport Chain (ETC). These ROS activate the PI3K/AKT pathway by inhibiting PTEN, a negative regulator. Consequently, this activation facilitates cell proliferation and survival, as well as the expression of hypoxia-related genes [24,25]. This study demonstrates that CAFs significantly increase ROS production in SiHa cells and overexpress genes important for hypoxia and glycolysis, including *ALDOC*, *ENO2*, and *PDK1*, which encode Aldolase C, Enolase 2, and Pyruvate dehydrogenase kinase 1, respectively.

The expression of the *ALDOC* and *ENO2* genes is strongly associated with the Warburg effect, which enables the activation of glycolysis and lactate production, even in a normoxic environment [26], whereas the function of PDK1 is to inactivate pyruvate dehydrogenase, the enzyme that converts pyruvate to acetyl-CoA [25], thus blocking the transition from glycolysis to oxidative phosphorylation in the mitochondria. In addition, PDK activity is associated with the upregulation of Hypoxia-Inducible Factor 1α (HIF-1α) [23], which activates the expression of genes involved in glycolysis, such as enolase, hexokinase, and lactate dehydrogenase, as well as the facilitated-diffusion glucose transporters GLUT1 and GLUT3 [27]. This strongly supports our results, in which SiHa cells, under CAF stimuli, undergo greater glycolytic activation and an increase in ROS, leading to increased survival of tumor cells. This evidence also explains why there were no significant differences in cell death, since the response is mainly directed toward the survival of tumor cells rather than apoptosis.

Furthermore, we found overexpression of genes related to migration and epithelial-mesenchymal transition in SiHa, including *TNC*, *CRLF1*, and *ENO2*; however, this does not align with the functional assay results, as CAF did not increase tumor cell migration. In addition, we identified other genes, such as *CXCL8*, *MYC*, *FOSB*, and *FOSL1*, that are involved in signaling via the NF-κB and AP-1 pathways and may directly impact tumor cell metabolism and survival. *MYC* is a gene favored by the PI3K/AKT/NF-κB pathway [28,29]. *FOSB* and *FOSL1* belong to the AP-1 family, a transcription factor family regulated by IL-8 (*CXCL8*), MAPK, and PI3K/AKT signaling [30].

On the contrary, we observed that HeLa cells respond differently to CAF stimuli, as mitochondrial activity does not vary with HG CAF, and ROS production shows no differences across any of the stimuli. This pattern is explained by the pathway with the greatest enrichment, TNF-α signaling via NF-κB. Although this cytokine was not found to be elevated, we did observe overexpression of the IL-8 gene (*CXCL8*), which is involved in activation of the NF-κB pathway, a transcription factor whose p50 and p65 subunits bind to the HIF-1α promoter, thereby activating the response to hypoxia [31]. In turn, HIF-1α promotes NRF2 expression, another transcription factor that regulates mitochondrial ETC components and contributes to their proper function, along with antioxidant proteins [32,33]. This clearly supports the absence of differences in mitochondrial activity and ROS levels. Likewise, it has been documented that HIF-1α activity, along with NF-κB, increases resistance to apoptosis [32], explaining why we did not find differences in CAF-induced apoptosis.

Moreover, we found that CAFs promote HeLa cell migration, a result that is reinforced by migration-related pathways such as epithelial-mesenchymal transition and KRAS signaling, in which the main genes that contribute to enrichment belong to ECM proteins like *COL6A3* (alpha-3 chain of type VI collagen), adhesion proteins such as *ITGB2* (β2 subunit of integrin), or genes that regulate migration, such as *EREG* (Epiregulin). EREG is an EGFR ligand that contributes to carcinogenesis through KRAS, increasing the migration of breast and bladder tumor cells, and it also acts in synergy with IL-6/JAK/STAT3 signaling to contribute to tumor progression [34]. This latter pathway is also enriched under CAF stimulation, indicating that it may promote tumor cell migration by activating the JAK/STAT3 and PI3K/AKT signaling pathways through its receptor IL-6R/gp130 [35].

Furthermore, IL-8 increases HeLa cell migration by inducing ERK phosphorylation and promoting expression of its own receptor, IL-8RA/IL-8RB, thereby creating a positive feedback loop [36]. In support of this, it has been demonstrated that IL-6 signaling induces IL-8 expression, and vice versa, via activation of the MAPK/AP-1 and PI3K/AKT/NF-κB signaling pathways [29,30,37]. This supports the idea that both cytokines are present at significantly elevated levels and therefore promote tumor cell migration.

Finally, we observed that in HeLa cells, CAFs promote IFN-γ production, whose effects are regulated by several enzymes, including SHP-1 phosphatase, encoded by *PTPN6*, a gene we found to be overexpressed. This gene has been associated with a poorer prognosis in CC because it promotes expression of indoleamine 2,3-dioxygenase 1 (*IDO1*) [38], which converts tryptophan to kynurenine, thereby reducing T cell function and fostering an immunosuppressive environment [39].

Notably, our work demonstrated that E2-stimulated CAFs did not differ from unstimulated CAFs in their effects. This suggests that, under our experimental conditions, exposure to E2 does not significantly alter their modulatory capacity toward tumor cells. However, E2 itself does have a direct impact on tumor cells, as demonstrated in previous studies [4,5,6,19]. Conversely, we hypothesize that the influence of E2 on CAFs may sustain a microenvironment exhibiting protumor properties, as GPER signaling in breast cancer CAFs promotes their proliferation, elevates the expression of the aromatase gene (*CYP19A1*), and enhances E2 production [22], thereby guaranteeing the continuous support provided by CAFs to tumor cells within the TME. Furthermore, considering that CAFs do not alter their cytokine production between low and high-grade lesions, this may imply that the activation of CAFs occurs at an early stage and is maintained throughout tumor progression.

## 5. Conclusions

In summary, this study reveals that CAFs modulate protumoral activities in a subtype-specific manner. While CAFs induce a metabolic reprogramming linked to hypoxia and glycolysis in squamous-cell carcinoma (SiHa), they promote a migratory and proinflammatory phenotype in adenocarcinoma (HeLa) via EMT, KRAS, and IL6/JAK/STAT3 signaling pathways. Moreover, the data suggest that CAF-induced alterations in CC cells are maintained regardless of E2 stimulation, with stromal activation appearing at early stages and remaining stable throughout tumor development. This establishes a foundation for exploring the functional reprogramming of CAFs as a supplementary therapeutic target to optimize clinical responses and patient well-being.

## Data Availability

The datasets used and/or analyzed during the current study are available from the corresponding author upon reasonable request.

## Supplementary Materials

The following supporting information can be downloaded at: https://www.mdpi.com/article/10.3390/cancers18101509/s1, Figure S1: Histological sections from low- and high-grade; Figure S2: Photographs from the primary culture of CAFs from low- and high-grade lesions.
