# Supplementary material for: Differential Protumoral Mechanisms Induced by CAFs in Cervical Cancer Cells Occur Independently of 17β-Estradiol Stimulation
Source: Cancers (Basel). 2026 May 8;18(10):1509. doi: 10.3390/cancers18101509 (PMC13204976; doi:10.3390/cancers18101509)
Supplement: Supplementary file 1 [file cancers-18-01509-s001.zip › cancers-4303983-supplementary.pdf]

Histological sections of low- and high-grade tumor tissues stained with hematoxylin and eosin (H&E). Hematoxylin stains nuclei in purple-blue and eosin stains cytoplasmic and stromal components in pink. Tumor cell nests are embedded within and surrounded by eosinophilic stromal areas (red arrows).

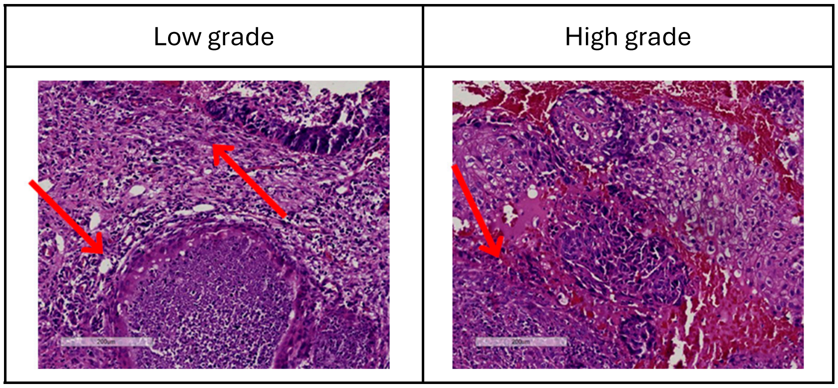

Supplementary Figure S1. Histological sections from low- and high-grade.

CAFs demonstrate a heterogeneous directional arrangement, stellate morphology, and overlapping proliferation (red arrows), consistent with previously published data on CAFs' behavior in primary culture.

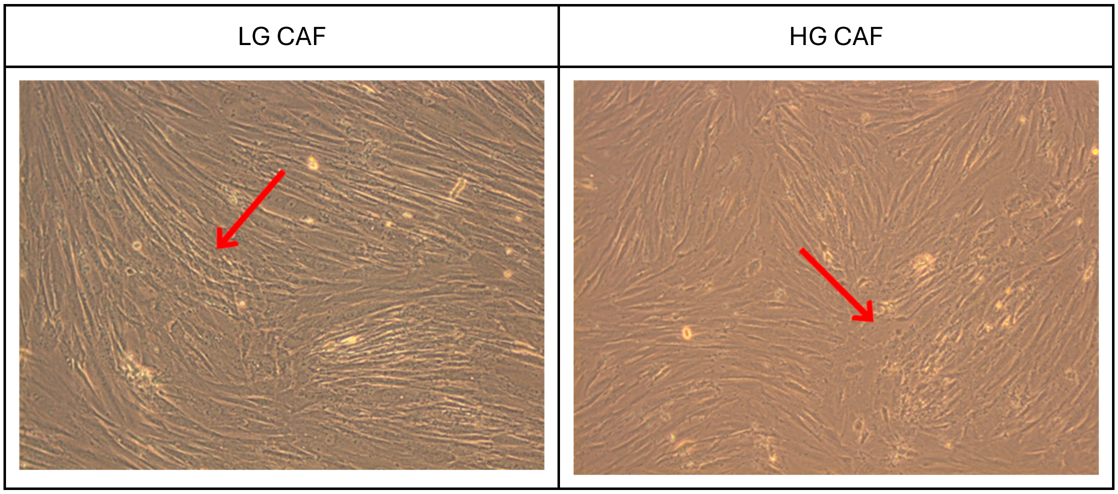

Supplementary Figure S2. Photographs from the primary culture of CAFs from low- and high-grade lesions. Images show the characteristic morphology and growth of CAFs.
